# Exploration and comparison of bacterial communities present in bovine faeces, milk and blood using 16S rRNA metagenomic sequencing

**DOI:** 10.1371/journal.pone.0273799

**Published:** 2022-08-31

**Authors:** Khethiwe Mtshali, Zamantungwa Thobeka Happiness Khumalo, Stanford Kwenda, Ismail Arshad, Oriel Matlahane Molifi Thekisoe

**Affiliations:** 1 Biomedical Sciences Department, Tshwane University of Technology, Pretoria, South Africa; 2 Unit for Environmental Sciences and Management, North-West University, Potchefstroom, South Africa; 3 Faculty of Veterinary Science, Department of Veterinary Tropical Diseases, University of Pretoria, Onderstepoort, South Africa; 4 Study Management, ClinVet International, Bainsvlei, Bloemfontein, South Africa; 5 Sequencing Core Facility, National Institute for Communicable Diseases, National Health Laboratory Service, Johannesburg, South Africa; 6 Faculty of Science, Department of Biochemistry and Microbiology, Engineering and Agriculture, University of Venda, Thohoyandou, South Africa; University of Illinois College of Medicine, UNITED STATES

## Abstract

Cattle by-products like faeces, milk and blood have many uses among rural communities; aiding to facilitate everyday household activities and occasional rituals. Ecologically, the body sites from which they are derived consist of distinct microbial communities forming a complex ecosystem of niches. We aimed to explore and compare the faecal, milk and blood microbiota of cows through 16S rRNA sequencing. All downstream analyses were performed using applications in R Studio (v3.6.1). Alpha-diversity metrics showed significant differences between faeces and blood; faeces and milk; but non-significant between blood and milk using Kruskal-Wallis test, *P* < 0,05. The beta-diversity metrics on Principal Coordinate Analysis and Non-Metric Dimensional Scaling significantly clustered samples by type (PERMANOVA test, *P* < 0,05). The overall analysis revealed a total of 30 phyla, 74 classes, 156 orders, 243 families and 408 genera. Firmicutes, Bacteroidota and Proteobacteria were the most abundant phyla overall. A total of 58 genus-level taxa occurred concurrently between the body sites. The important taxa could be categorized into four potentially pathogenic clusters *i*.*e*. arthropod-borne; food-borne and zoonotic; mastitogenic; and metritic and abortigenic. A number of taxa were significantly differentially abundant (DA) between sites based on the Wald test implemented in DESeq2 package. Majority of the DA taxa (*i*.*e*. *Romboutsia*, *Paeniclostridium*, *Monoglobus*, *Akkermansia*, *Turicibacter*, *Bacteroides*, *Candidatus_Saccharimonas*, *UCG-005* and *Prevotellaceae_UCG-004*) were significantly enriched in faeces in comparison to milk and blood, except for *Anaplasma* which was greatly enriched in blood and was in turn the largest microbial genus in the entire analysis. This study provides insights into the microbial community composition of the sampled body sites and its extent of overlapping. It further highlights the potential risk of disease occurrence and transmission between the animals and the community of Waaihoek in KwaZulu-Natal, Republic of South Africa pertaining to their unsanitary practices associated with the use of cattle by-products.

## Introduction

Livestock rearing plays a vital role in sustenance of the livelihoods of rural communities [1]. However, livestock may serve as a potent reservoir of different pathogenic organisms that could have devastating health and economic implications, especially when proper husbandry and hygiene practices are not in place [2]. It may additionally have compounding effects on the public health due to the zoonotic nature of some of the associated diseases [3].

Cattle products and by-products are utilized to provide nourishment and income; play important social roles in many cultural traditions; and help to facilitate day-to-day household activities [2,4]. Bovine faeces, milk and blood are no exception and they are among the most commonly used cattle by-products particularly by the targeted rural community. Briefly, fresh faeces are used to seal and insulate the clay plastered floors inside the homes; applied as an anti-microbial agent to heal wounds and consumed as an anti-diarrheal medicine; it may be formed into dung ‘cakes’ which are dried up and used to fuel cooking fire; while aged or composted manure is utilized as a fertilizer for the crops. The milk obtained from the cattle in this community is often consumed raw, boiled or fermented to make sour milk which is mostly eaten by vulnerable groups such as children and the elderly. They occasionally use milk to cure a number of ailments *e*.*g*. as a natural laxative and for its opposite anti-diarrheal effect. On the other hand, blood from freshly slaughtered animals may be smeared on parts of the body or drunk raw during ritualistic feasts. It may also be cooked into a broth and consumed as a delicacy. This knowledge is acquired through tribal affiliation of the first author, nonetheless these are age-old traditions and rituals practiced among different African tribes and rural communities of the world for more or less similar reasons and have been recorded by a number of authors [5–7]. The above-mentioned ‘unsanitary’ practices including rearing of livestock in close proximity to households poses a major risk of disease transmission between animals and humans as previously expounded [3,8–11].

From an ecological perspective, the mammalian body is considered to be a complex ecosystem consisting of various interconnected ecological niches *i*.*e*., body sites [12]. Although each body site harbours distinct and specialized microbiota, there is an interplay of factors which influence the microbial colonization and assembly [12]. According to Rainard [13] explorations of the complex microbial communities within the different body sites might contribute to knowledge of disease occurrence and improvement of health and livestock productivity.

Development and improvement of next generation sequencing (NGS) and other omics technologies in the last decade has allowed the study of host-associated microbial communities in mammals, particularly ruminants, at a depth never before possible [14]. Most mammalian studies using these technologies have mainly dealt with exploration of the skin, mouth and gut microbiota but other body sites are now increasingly considered as harbouring their own microbiota, for instance, the lungs; oro-pharyngeal, urinary and genital tracts [13,15]; mammary glands [12,16,17] and more recently the bloodstream [18–20].

Chiefly, NGS-based bovine investigations have been focussed on the microbial community of the rumen due to its importance in feed efficiency and contribution to milk and meat production [14,21–25]. There is also a rise in the number of studies reporting on microbial profiling of bovine faeces using such technologies [18,20,25–27], mostly supplementing the ruminal microbiome studies.

The NGS-based investigations of milk have been primarily conducted on secretions of clinical vs subclinical mastitic quarters and in some instances compared to those of healthy quarters [12,16,28–35]. The focus has mainly been on how the microbial flora of milk changes when it becomes a food product [15], but rarely have these studies been conducted to discern the global diversity of the milk microbiome in relation to animal health and physiology [12,15].

Similarly, the majority of published research conducted on bovine blood has been focused on detection of selected pathogens of veterinary significance that lead to production and economic decline rather than the microbiome of animals in relation to their health and physiological state. The few studies that explore the blood microbiome of cattle through NGS have focused on investigations of its role in the endogenous entero-mammary [18] and haematogenous [20] translocation of gut or uterine microbes.

Research conducted on mice and human breast milk suggests a presence of an endogenous entero-mammary pathway where live bacteria can be transferred from intestines through intestinal dendritic cells and macrophages to mammary glands via lymphatic and peripheral blood circulation [12,13,15,18,36]. Although not providing compelling evidence of this pathway in bovines, a study of milk somatic cells, bloodstream macrophages and faeces reported on simultaneous detection of bacterial signatures in the three body sites [18]. This postulated pathway has however been met with great disdain by some authors [12,13] who dispel its existence in ruminants and provide alternative theoretically driven and hypothetical pathways by which the gut bacteria find their way to the mammary glands.

In this study we primarily aimed to explore and compare the microbial community structure and taxonomic composition of faecal, milk and blood samples collected from lactating cows. Secondarily, we aimed to identify potentially pathogenic taxa of veterinary and / or medical significance present between the three sample groups and subsequently assess the potential repercussions of the ‘unsanitary’ practices pertaining to the use of cattle by-products by the small-scale subsistence farmers of Waaihoek community in KwaZulu Natal.

The aims were achieved through exploratory experimentation, using high-throughput 16S rRNA metagenomic sequencing on the Illumina Miseq platform. Furthermore, our study presents novel insights into the microbiota of bovine faeces, milk and blood in the Republic of South Africa (RSA) using amplicon sequence variants (ASVs) inferred through the high-resolution Divisive Amplicon Denoising Algorithm 2 (DADA2) pipeline.

## Materials and methods

### Animal sampling

The study was approved by the North West University’s Faculty of Natural and Agricultural Sciences Research Ethics Committee (NWU-01757-20-A9) and was conducted following the guidelines of the institutional Animal Care, Health and Safety Research Ethics Committee (NWU-AnimCareREC). Sampling was conducted at 8:00 am on the 17^th^ of April 2019 at the Niekerskraal cattle dip site in Waaihoek village, situated on the outskirts of Ladysmith in KwaZulu-Natal, RSA (GPS coordinates: -28.46822280; 30.0880990). A verbal agreement to an informed consent to participate in the study was obtained from the animal owners and / or herders present at the communal dip site. The cows were not placed on any special diet prior to sampling however, the owners and herders confirmed that their daily diet is typically constituted of kitchen left overs in the morning and during the day they are allowed to roam in search of forage, either supervised or unsupervised. At night they are housed in kraals situated in close proximity to the homes of the owners. Prior to sampling the cows were rested and allowed to forage on the overgrown thatch grass surrounding the dip tank and crush pens. The sampling population included n = 110 mixed breed cows, *i*.*e*. 33 lactating and 77 non-lactating which were representative of the entire cow population owned by the Waaihoek community utilising the cattle dip.

Three sample sets (*i*.*e*. faeces, milk and blood) were collected per lactating cow while only blood samples were collected from the non-lactating cows. This was achieved through the aid of certified Animal Health Technicians from uThukela Veterinary Services. All sampling was conducted aseptically while the cows were restrained in crush pens. Briefly, faecal samples were retrieved directly from the rectum using gloved hands. The gloves were replaced with every new evacuation and each handful of faecal sample was placed into a sterile zip-lock bag [37]. For milk collection, individual teat surfaces were scrubbed clean with moist cotton balls, impregnated with 70% ethanol for 10 to 15 s. Composite milk samples were collected into sterile 15 mL vials from the pre-cleaned teats, discarding the first three streams per teat as previously described [38]. Blood samples were collected targeting the tail ventral midline groove of the cows. The site was swabbed clean in a similar manner to the teats prior to venipuncture. Blood was drawn into sterile 4 mL EDTA coated vacuum tubes using sterile 21 gauge, 25 mm hypodermic needles [39]. The collected samples were transported to the laboratory in separate coolers containing ice packs, thereafter pre-processed for DNA extraction and stored in the -80°C freezer until further use.

### DNA extraction, pooling and quantification

Extraction of DNA from the pre-processed samples was conducted at the Molecular Parasitology and Zoonosis Research Group laboratory located at the Unit for Environmental Sciences and Management, North-West University, Potchefstroom Campus, RSA. Faecal microbial DNA was extracted using the Quick-DNA^TM^ Fecal/Soil Microbe extraction kit (Zymo Research, Inqaba Biotec^TM^) from ≤ 150 mg of faecal samples. The faecal samples were lysed by bead beating in a ZR BashingBead™ lysis matrix filled with buffer and placed in a bead beater (TissueLyser LT, Qiagen^®^) prior to DNA extraction. On the other hand, milk and blood microbial DNA were extracted from buffer lysed 200 μL of samples using the Quick-DNA^TM^ Miniprep kit (Zymo Research, Inqaba Biotec^TM^). The eluted DNA samples were quantified using Qubit^®^ Fluorometer 4.0 (Invitrogen, Thermo Fisher Scientific Inc) and stored at -20°C for downstream molecular application.

For 16S rRNA sequencing, sample sets from two animals were excluded due to poor visual quality of the milk. Therefore, DNA samples extracted from corresponding faecal, milk and blood samples of 31 lactating cows were sequenced. The samples were pooled according to farm origin (*i*.*e*. A-D, based on the farmer’s identity) in random groups of three, with the exception of the sample set obtained from an animal with a retained placenta. Each pooled DNA sample of either faeces, milk or blood was therefore constituted of an equimolar mixture of three samples obtained from three different animals. Following initial random assignment DNA samples from the same group of animals were strategically matched across the three sample groups. The pools were quantified, then normalized for Illumina sequencing. For *Anaplasma* prevalence determination, DNA was extracted from a total of n = 110 blood samples obtained from both lactating and non-lactating cows.

### Amplification of bacterial 16S rRNA and sequencing

The V3-V4 hypervariable region of the bacterial 16S rRNA gene was amplified using universal bacterial primers: PCR Forward Primer: 5′ GTC TCG TGG GCT CGG AGA TGT GTA TAA GAG ACA GGA CTA CHV GGG TAT CTA ATC C 3′ and PCR Reverse Primer: 5′ TCG TCG GCA GCG TCA GAT GTG TAT AAG AGA CAG CCT ACG GGN GGC WGC AG 3′ [Integrated DNA Technologies, Whitehead Scientific (Pty) Ltd]. In the laboratory, samples were aseptically processed to minimise contamination. Two no template controls (NTCs) *i*.*e*., NSCF-neg1 and NSCF-neg2 consisting of PCR and sequencing laboratory reagents as well as nuclease free water in place of experimental DNA template were incorporated in the amplification and sequencing steps and processed alongside the experimental samples. Library preparation was performed according to the standard instructions of the 16S Metagenomic Sequencing Library preparation protocol (Illumina^TM^, Inc., San Diego, CA, United States). Indexed amplicons were quantified using Qubit^®^ High Sensitivity dsDNA Assay Kit (Thermo Fisher Scientific) and the sizes of the amplicons were visualised using the 4200 TapeStation (Agilent Technologies, Germany). The normalized libraries were pooled for sequencing, denatured to single strand using NaOH, then PhiX (10%) was added to the library. Libraries were then sequenced using the MiSeq Reagent kit v3 (Illumina^TM^ Inc., San Diego, CA, USA) and paired-end 2 × 300 bp sequencing was performed at the Sequencing Core Facility of the National Institute for Communicable Diseases, RSA. The generated sequences from the NTCs were compared to sequences of experimental samples in the retrospective assessment of contamination step.

For prevalence determination of *Anaplasma* species among the cow population following the results of community analysis, PCR was conducted using the 16S rRNA primer pairs (fD1: 5’AGAGTTTGATCCTGGCTCAG3’; rP2: 5’ACGGCTACCTTGTTACGACTT3’) with an expected fragment size of 1470 bp [40]. The PCR reaction was performed in a total reaction volume of 25 μL which contained 12,5 μL of the 2X Kapa HiFi Hotstart ReadyMix (Kapa Biosystems, Roche), 1 μL of each of the forward and reverse primers (2 μM concentration) and 3 μl of DNA template. The thermal cycling conditions included an initial denaturation step of 98°C for 10 sec, followed by 35 cycles of denaturation at 98°C for 1 sec; annealing at 55°C for 5 sec and extension at 72°C for 15 sec, terminating with a final extension step at 72°C for 1 min held at 4°C ∞. The PCR products were size fractionated in 1,5% agarose gel stained with ethidium bromide, the amplicons were verified using confirmed *A*. *centrale* and *A*. *marginale* positive controls and photographed under UV transillumination (Enduro^TM^ GDS gel documentation system, Labnet International Inc.). PCR positive samples were Sanger sequenced at Inqaba Biotec^TM^ (RSA) and the sequences were confirmed with those deposited on the GenBank database of the National Center for Biotechnology Information (NCBI).

### Bioinformatics and statistical analyses

The generated paired-end sequences, in FASTQ format, were initially processed using FastQC (v0.11.8) and trimGalore (v0.6.4_dev; https://github.com/FelixKrueger/TrimGalore) for quality control (including determining the sequence base content, Kmer frequency, GC content, sequence length, duplication and adapter contamination) and filtering (which included adapter removal and read trimming), respectively. Only reads with a quality score of 20 or higher (Q ≥ 20) and at least a length of 50 base pairs (≥ 50 bp) were considered for downstream analysis.

All the downstream analyses were performed in R (v3.6.1) within R studio. Clean reads were pre-processed using the high-resolution Divisive Amplicon Denoising Algorithm 2 (DADA2) package (v1.12.1). Amplicon Sequence Variants (ASVs) were inferred as previously described [41]. Taxonomy was assigned to the obtained ASVs to species level and the ASV abundance estimates were determined using SILVA SSU taxonomic training data formatted for DADA2 (v138, 99% 16S full-length; [42], https://zenodo.org/record/3986799#.YG7YR-gzY2w). ASVs assigned as Archaea and Eukaryota were filtered out and further analyses were conducted only on Bacteria. Additional filtering and denoising was conducted in PhyloSeq package (v1.28.0) as described by McMurdie and Holmes [43]. Stacked bar plots of taxa present within and between samples at phylum and genus levels were plotted in Microsoft Excel (Windows 10).

Alpha (α)-diversity indices (*i*.*e*. Chao1, Shannon and Simpson’s) were estimated using the plot_richness function from PhyloSeq and mean comparison *P*-values calculated using stat_compare_means function from the ggpubr package (v0.4.0) and plotted with ggplot2 v3.2.1 [44]. The significant difference between groups was inferred using Kruskal-Wallis test, with *P*-values < 0,05 deemed significant. Effect sizes of the differences between groups were calculated using the Cohen’s D measure using the effsize package in R (https://github.com/mtorchiano/effsize), based on Shannon diversity indices.

Ordinations for β-diversity between groups were estimated using Principle Coordinate Analysis (PCoA) based on weighted-UniFrac distance and Non-Metric Multidimensional Scale (NMDS) using Bray distance metric as implemented in the plot_ordination and amp_ordinate functions in PhyloSeq and the ampvis2 package (https://madsalbertsen.github.io/ampvis2/articles/ampvis2.html), respectively. Permutational Multivariate Analysis of Variance (PERMANOVA) using permutation test with pseudo *F* ratios as implemented in the Adonis function of the Vegan R package (https://github.com/vegandevs/vegan) was used to determine significance for sample clustering on ordination plots. Similarly, *P*-values < 0,05 were deemed statistically significant. Sample groups were used as independent variables and taxa prevalences at ASV (for α-diversity) and genus (for β-diversity) levels as dependant variables.

Differential abundance analysis was performed using the negative binomial model (Wald test) implemented in DESeq2 v1.30.1 as previously described [45]. The normalized (log2-fold-change) data was ordered into a table by the adjusted *P*-value (*Padj* or q-value) according to the ASVs that were among the most significantly differentially abundant (DA) between sample types. The following pairwise combinations were used in the analysis: Blood vs Faeces, Blood vs Milk and Faeces vs Milk. The results were then plotted using ggplot2. The ASVs with *Padj* < 0,01 were regarded as significantly DA. Positive log2-fold change indicated increased abundance, while negative log2-fold change indicated decreased abundance for differential abundance plots.

UpsetR v1.4.0 was used to construct intersection plots depicting the shared and unique bacterial families and genera between the different sample groups [46].

## Results

### Characteristics of the population and sequencing

The 16S rRNA metagenomics study was conducted on samples obtained from lactating cows. The cows belonged to four small-scale subsistence farmers and they were mostly apparently healthy and no signs of udder inflammation were observed except in one animal which also had a retained placenta following a spontaneous abortion. Initially, a total of n = 99 samples were obtained from 33 animals, comprised of three sets of samples per cow, *i*.*e*. n = 33 faeces (assigned the label WF); n = 33 milk (WM) and n = 33 blood (WB). After pooling, a total of 33 DNA samples were sequenced using the Illumina Miseq sequencing platform (*i*.*e*. n = 11 faeces; n = 11 milk; and n = 11 blood). Post-sequencing, results of three DNA pool sets were excluded due to poor sequencing depth of the generated sequences. Eventually, sequence results from only 22 animals that passed quality filtering could be used in the structure and community analyses (*i*.*e*. n = 8 faeces; n = 8 milk; and n = 8 blood).

During retrospective assessment of contamination, the NTCs contained sequences corresponding to seven microbial genera. These included *Escherichia/Shigella Pseudomonas*, *Bacillus*, *Ralstonia*, *Blautia*, *Anaerobacillus* and *Lawsonella*. The sequences of three microbial genera (*Escherichia/Shigella*, *Pseudomonas* and *Bacillus*) were shared between the NTCs. These were also present in some of the experimental samples however, each microbial taxon is represented by multiple ASVs and those that matched the sequences within the NTCs were variably present and far fewer in number to constitute contamination. Therefore, at our discretion a decision was taken to retain all sequences in the analysis as true biological signals. Of the four remaining genera, two were uniquely present in NSCF-neg1 and two also in NSCF-neg2, none of which could be detected in the experimental samples. The NTCs were subsequently removed and not included in the community analyses.

An overall total of 602 011 (minimum = 925; mean = 25 084; maximum = 58 202; Standard Deviation = 16 836) non-chimeric Illumina reads were generated from the V3-V4 hyper-variable region of 24 pooled DNA samples, with a mean sequence read length of 430 bp (minimum = 251 bp; maximum = 468 bp). The number of reads including mean number ± standard error of the mean (SEM) obtained per group equalled 355 395 (44 424 ± 2591) in faeces, 156 257 (19 532 ± 4306) in milk and 90 359 (11 295 ± 2581) in blood. The number of reads retained across each step tracked through the DADA2 pipeline per sample pool are shown in S1 Table.

**Fig 1:**
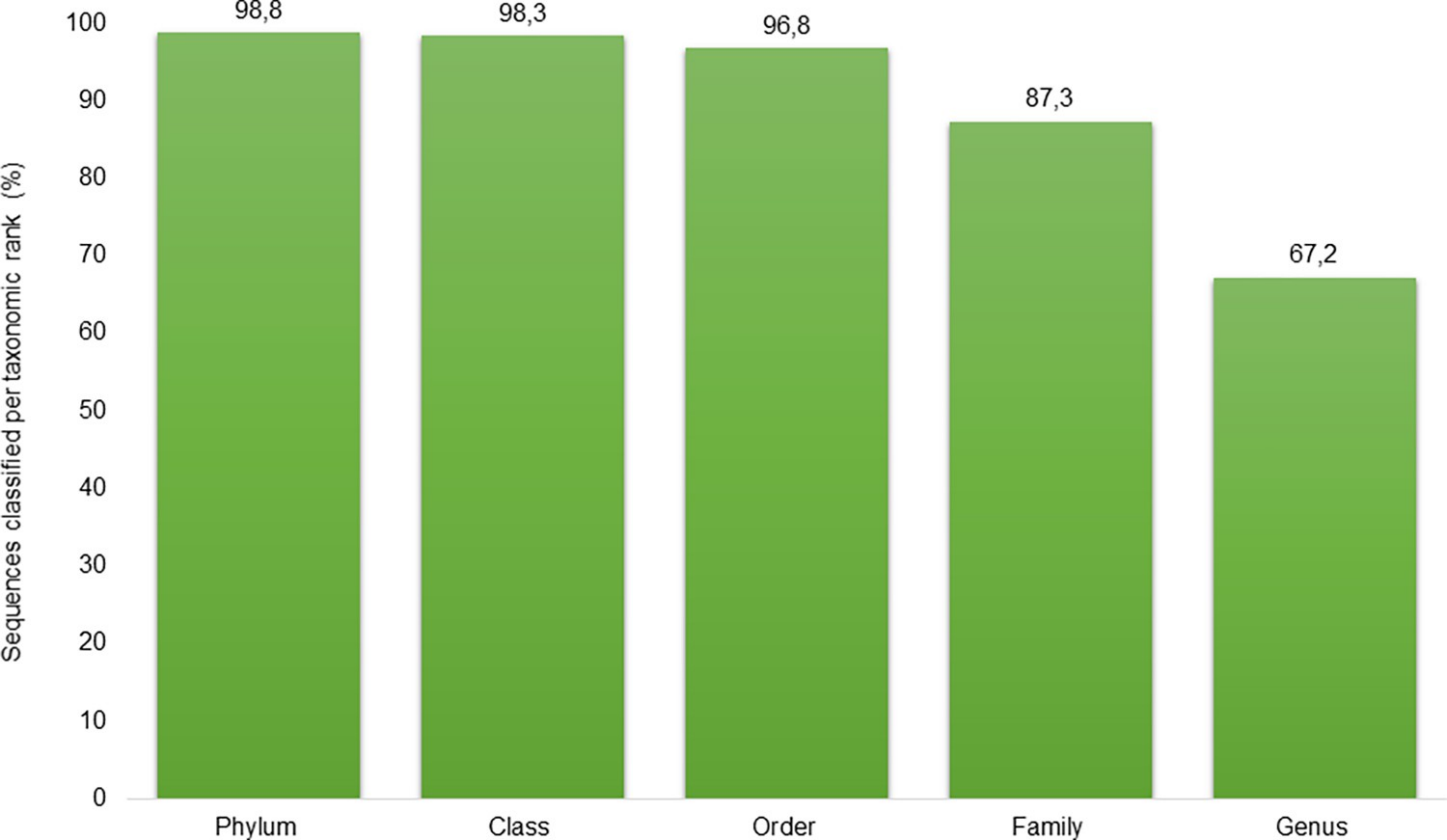
The proportion of amplicon sequence variants (ASVs) assigned at a given taxonomic rank using the SILVA database v138.

The reads obtained from the three sample groups were assembled into 8426 distinct ASVs at Kingdom (Bacteria) level through DADA2. At least 98,8% of the sequences could be assigned to a known phylum, with the proportion of assignments decreasing at lower taxonomic levels (Fig 1). Several taxa were assigned multiple ASVs and overall they were collapsed into a minimum of 5 (5WB) and maximum of 331 (6WM) ASVs representing unique taxa per sample pool. A minimum of 4 and maximum of 207 of the taxa contained per sample pool were assigned at genus level taxonomy (S1 Table).

Overall, a total of 67,2% of the taxa could be resolved to genus level, while 28,5% could not. The remaining 4,3% at this level was unaccounted for. The highest resolution of taxa at genus level was obtained in milk (67%), followed by faeces (64%) then blood (62%). Only three ASVs were tentatively resolved at species level among the body sites *i*.*e*. *Fusobacterium necrophorum*, *Luteimonas composti* and *Romboutsia sedimentorum*, accounting for 0,027% of the species that could be detected.

### Microbial diversity of bovine faeces, milk and blood

When analysing differences between the three sample groups, the alpha diversity box-plots reflected the minima, median, degree of dispersion, maxima and outliers of microbial diversity within groups (S1A–S1C Fig). The alpha diversities were estimated through Chao1 index, which measures richness and the Simpson’s and Shannon indexes, which combine both richness and evenness [47]. The effect size measurements (S2 Table) showed that the differences between the sample groups were large enough for us to assess relevant differences between microbial communities present in the three groups under study. The microbial communities from the faecal samples had significantly higher alpha diversity values than milk and blood samples as determined through the above-mentioned index estimators (Kruskal-Wallis: *P* = 8,1E-04, *P* = 0,0031 and *P* = 0,001, respectively). Nonetheless, all the obtained values between the three sample groups were statistically significant (*P* < 0,05). With pairwise analysis, the α-diversity varied significantly between faeces and blood groups based on Chao 1 (*P* = 1,6E-04), Shannon (*P* = 1,6E-04) and Simpson’s (*P* = 3,1E-04) index estimators. The same was observed between faeces and milk on Chao1 (*P* = 0,0047) and Shannon index estimators (*P* = 0,01). In contrast, there was no significant difference between blood and milk microbial communities via any of the used index estimators.

**Fig 2:**
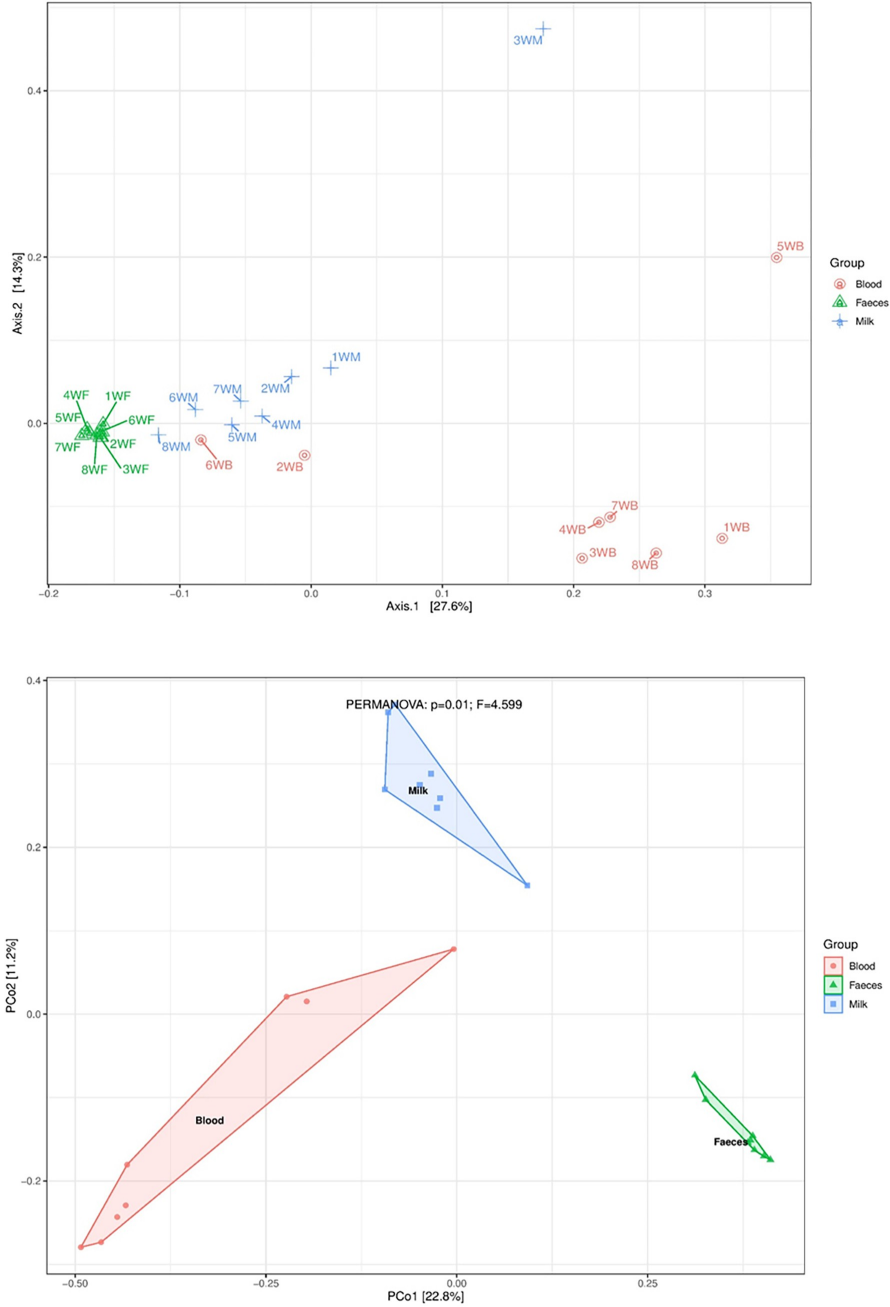
A: β-diversity of faecal (WF), milk (WM) and blood (WB) samples shown with Principal Coordinate Analysis (PCoA) based on weighted UniFrac metric, calculated using normalized data. The letter W denotes the sampling area, Waaihoek while the letters F, W and B denote sample type. Each point represents a sample pool. B: β-diversity shown with Non-Metric Dimensional Scaling plot (stress = 0.113) using Bray dissimilarity metric between sample groups. The sample groups are color coded. Significant differences: *P* = 0.01, *F* = 4.599, PERMANOVA.

To compare whole microbial composition dissimilarity between sample groups, PCoA and NMDS plots were analysed. Generally, the plots showed clear clustering of microbial communities by sample group with a few outliers (Fig 2A and 2B). Using the weighted UniFrac distance metric on PCoA which takes into account abundance and the phylogenetic distance between ASVs [48], three clusters by sample group could be observed (Fig 2A). The blood group was divergent from the faecal and milk groups however, the milk and faecal groups clustered quite closely with the first two principal coordinate axes accounting for 41,9% of the total dissimilarity among the samples.

The NMDS plot based on Bray distance measure showed similar findings to those calculated using PCoA; however, this metric provides a measure of community composition differences between samples based on ASV counts, regardless of taxonomic assignment [49]. In addition, the variances observed in the milk microbiota were significantly higher than the variances observed in either the faecal or blood microbiota. The differences in community structure between samples were statistically significant (Fig 2B, PERMANOVA: *P* = 0.01; *F* = 4.599, plot stress = 0.113). Conversely, this method showed exceptionally clear clustering between the sample groups than PCoA.

### Microbial composition of bovine faeces, milk and blood samples

**Fig 3:**
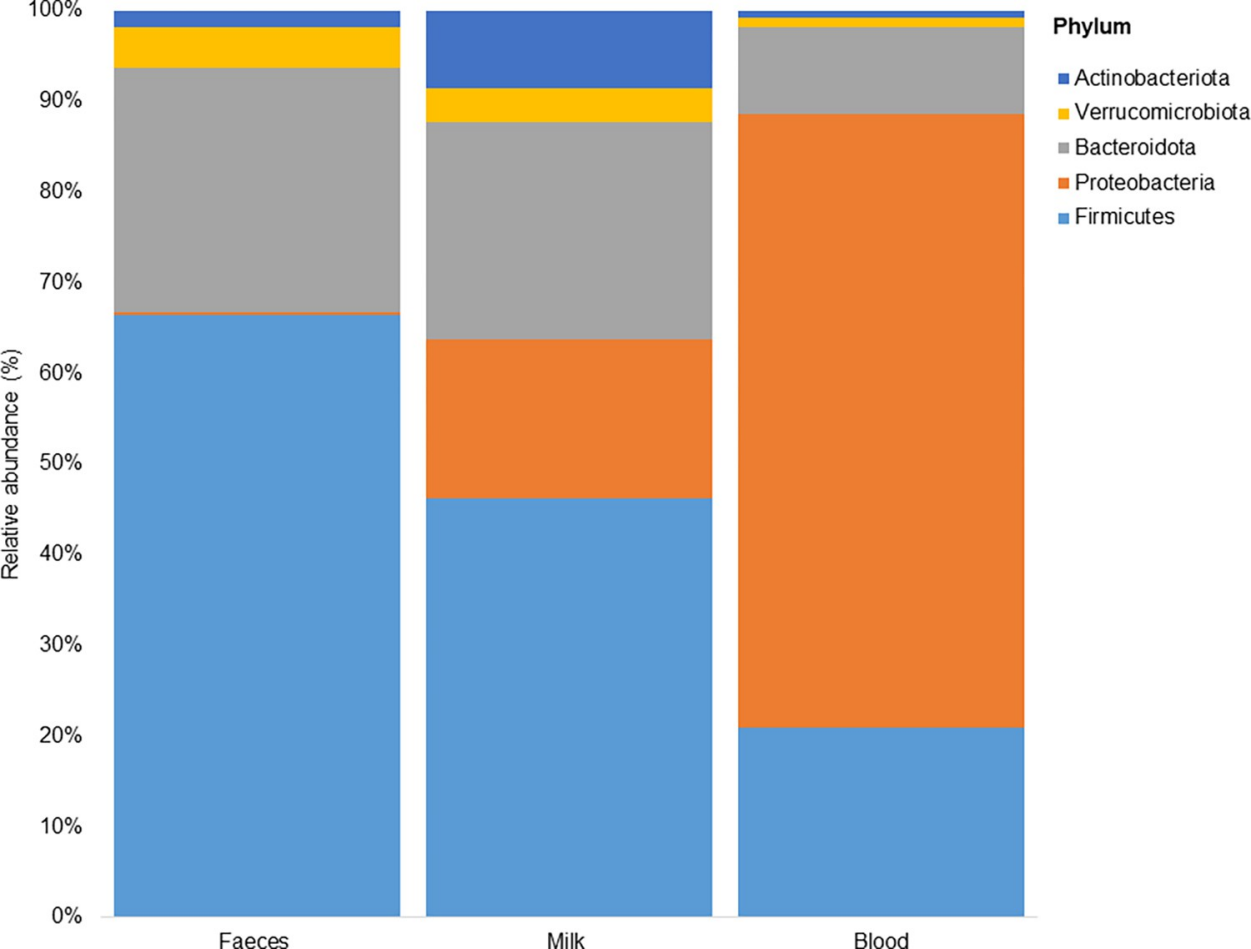
Stacked bar-plot with proportions of bacterial phyla detected from the three sample groups. Relative abundance graphed along the y-axis and sample type along the x-axis.

**Fig 4:**
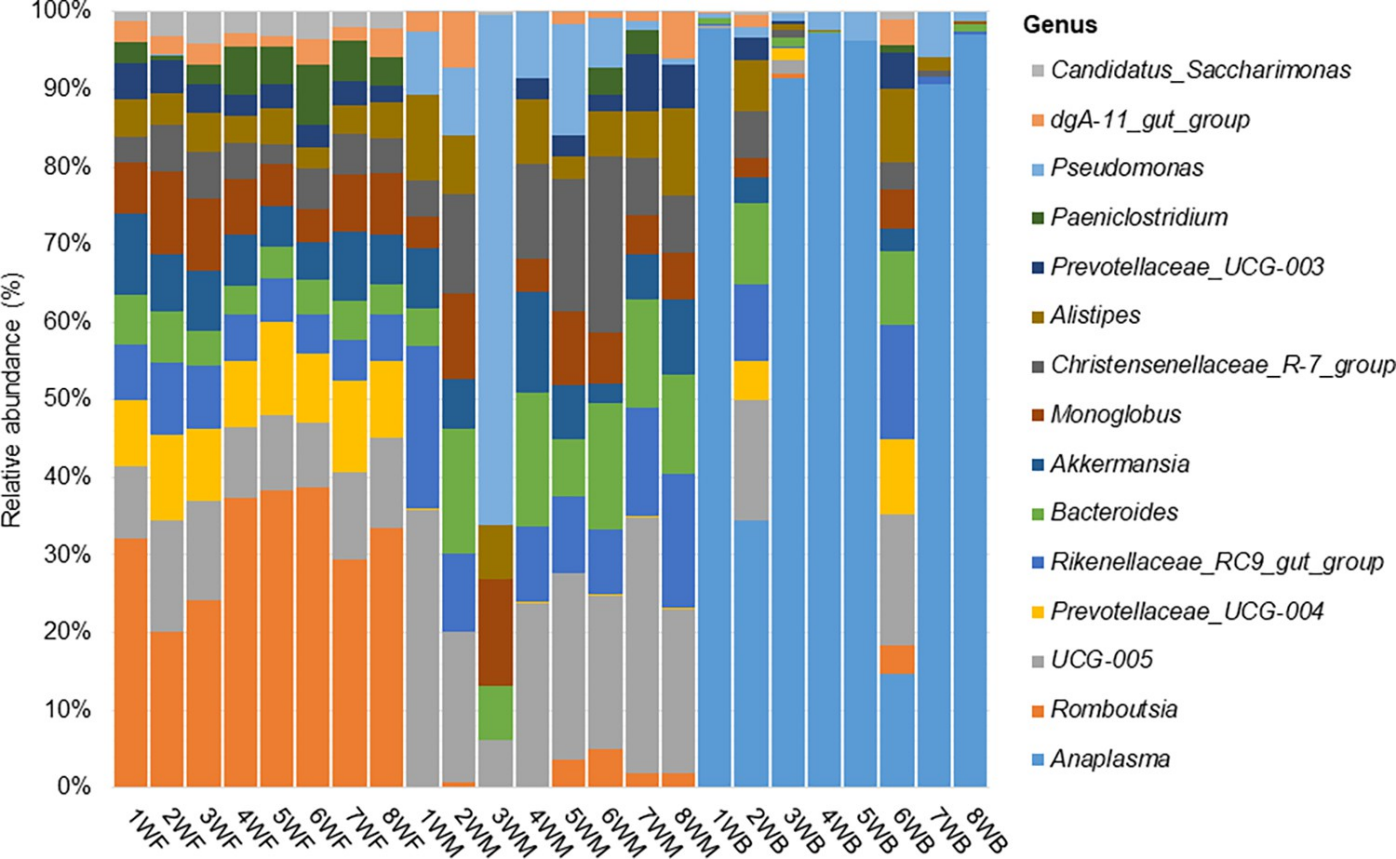
Distribution of the 15 most abundant genus-level taxa across faecal, milk and blood samples. Relative abundance graphed along the y-axis and sample type along the x-axis.

**Fig 5:**
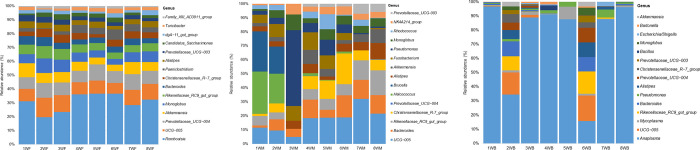
A. Distribution of the top 15 genus-level taxa averaged between the faecal samples. Relative abundance graphed along the y-axis and sample type along the x-axis. B: Distribution of the top 15 genus-level taxa averaged between the milk samples. Relative abundance graphed along the y-axis and sample type along the x-axis. C: Distribution of the top 15 genus-level taxa averaged between the blood samples. Relative abundance graphed along the y-axis and sample type along the x-axis.

Taxonomy bar charts were created to note differences and similarities among bacterial taxonomic ranks within and between the sample groups (Figs 3–5A and 5B). An overall total of 30 bacterial phyla were obtained with the highest number (30) detected in milk samples. Despite the high alpha diversity, the least number of phyla (14) was recorded from faecal samples, surpassed by blood samples which contained 18 phyla. The five most abundant phyla detected across the three body sites were Firmicutes (42,5%), Proteobacteria (25,6%), Bacteroidota (18,8%), Verrucomicrobiota (3,0%) and Actinobacteriota (3,0%) in varying group abundances (Fig 3). These accounted for 97,6%; 85,3%; and 98,3% of the bacterial communities contained in faeces, milk and blood, respectively. Firmicutes and Bacteroidota predominated faecal samples (at 64% and 25,9% relative abundances, respectively); similarly milk samples (at 39,4% and 20,4% relative abundances, respectively). In contrast, blood samples were predominated by Proteobacteria (66,4%) and Firmicutes (20,6%). A considerable proportion of Verrucomicrobiota and Actinobacteriota were detected in milk (3,2% and 7,3%, respectively) and faeces (4,4% and 1,7%, respectively) but less abundantly in blood (1,2% and 0,7%, respectively). Milk also contained Proteobacteria (15,0%), while faeces contained a negligible amount of this phylum (0,2%).

Overall, 74 classes were resolved across the three sample groups, the majority (74) were detected in milk samples and the least in faeces (21). A total of 156 orders could be resolved, while 243 families and 408 taxa representing genera were resolved between the three sample groups (S3 Table). The highest number of taxa in the afore-mentioned rankings were identified in milk (154 orders, 236 families and 374 genera), whereas the lowest count of taxa were observed in faeces (38 orders, 55 families and 98 genera).

The distribution of the top 15 most abundant genus-level taxa (average relative abundance = 3,4%) across the samples is shown on Fig 4, their respective rankings (1 = most abundant; 15 = least abundant) across the three body sites are indicated in S4 Table. These taxa contributed 51,6% to the overall abundance of the taxa assigned at genus level and 83,2%; 34,7%; and 87,9% to the respective faecal, milk and blood groups. The taxa were predominated by members of the phylum Bacteroidota (40%), followed by Firmicutes (33%). The largest genera by relative abundance included *Anaplasma* (19,4%), *Romboutsia* (7,4%), *UCG−005* (4,4%) and *Prevotellaceae_UCG−004* (3,1%).

When investigating the distribution patterns of microbial taxa across the three body sites, it was observed that the prevalent taxa were diverse. However, some genera such as *UCG-005* (2,5–8,9%), *Rikenellaceae_RC9_gut_group* (2,0–5,4%), *Bacteroides* (1,8–4,1%), *Alistipes* (1,3–3,4%), *Prevotellaceae_UCG-004* (1,2–8,3%), *Christensenellaceae_R-7_group* (0,8–3,9%), *Prevotellaceae_UCG-003* (0,6–2,8%), *Monoglobus* (0,5–6,0%) and *Akkermansia* (0,4–6,0%) were among the prevalent taxa across the three body sites. The range in relative abundances of the taxa across the sample groups are given in parentheses, with the lowest abundances corresponding to the blood group, while the highest abundances corresponded to the faecal group.

It could also be observed that bacterial profiles from milk more closely resembled those from faeces in terms of contained taxa and proportions of the 15 most abundant genus-level taxa, corroborating the findings from β-diversity analysis. Although blood samples also possessed similar taxa, they were in much lower proportions in comparison to milk and faecal samples and their distribution was unique across individual blood sample pools.

The core microbial taxa found in bovine faeces, milk and blood were catalogued as genus ranking taxa that were consistently present among ≥ 75% of all samples per respective group with an overall relative group abundance of ≥ 0,1%, listed in descending order of sequence abundance (Table 1).The number of cows that were positive for each genus, the average and the range of the total population represented by each genus across all sampled cows are also shown on the table.

**Table 1:** Core microbiota present in ≥ 75% of samples per group at ≥ 0,1% relative abundance including average and range across cow samples.

	Genus-level taxa	Number of sequences per genus	Relative abundance per group (%)	Number of samples positive out of 8 (%)	Average and (range) across cow samples (%)
**Faecal core microbiota**	*Romboutsia*	25687	26,6	8 (100)	18,6 (10,7–24,3)
	*UCG-005*	8553	8,9	8 (100)	6,2 (5,2–7,7)
	*Prevotellaceae_UCG-004*	7959	8,3	8 (100)	5,8 (5,0–7,2)
	*Akkermansia*	5770	6,0	8 (100)	4,2 (3,1–6,4)
	*Monoglobus*	5770	6,0	8 (100)	4,2 (2,6–5,7)
	*Rikenellaceae_RC9_gut_group*	5198	5,4	8 (100)	3,8 (3,0–4,9)
	*Bacteroides*	3863	4,0	8 (100)	2,8 (2,1–3,9)
	*Christensenellaceae_R-7_group*	3728	3,9	8 (100)	2,7 (1,5–3,3)
	*Paeniclostridium*	3437	3,6	8 (100)	2,5 (0,3–4,9)
	*Alistipes*	3289	3,4	8 (100)	2,4 (1,7–3,0)
	*Prevotellaceae_UCG-003*	2653	2,8	8 (100)	1,9 (1,2–2,9)
	*Candidatus_Saccharimonas*	2187	2,3	8 (100)	1,6 (0,8–2,3)
	*dgA-11_gut_group*	1956	2,0	8 (100)	1,4 (0,9–2,0)
	*Turicibacter*	1871	1,9	8 (100)	1,3 (0,4–2,4)
	*Family_XIII_AD3011_group*	974	1,0	8 (100)	0,7 (0,3–1,1)
	*NK4A214_group*	956	1,0	8 (100)	0,7 (0,5–0,8)
	*Clostridium_sensu_stricto_1*	905	0,9	8 (100)	0,7 (0,1–1,5)
	*Treponema*	766	0,8	8 (100)	0,6 (0,3–0,9)
	*DNF00809*	718	0,7	8 (100)	0,5 (0,4–1,1)
	*Olsenella*	653	0,7	8 (100)	0,5 (0,2–0,8)
	*Candidatus_Soleaferrea*	624	0,6	8 (100)	0,5 (0,2–0,7)
	*Prevotellaceae_UCG-001*	608	0,6	8 (100)	0,4 (0,2–0,6)
	*Coprococcus*	606	0,6	8 (100)	0,4 (0–0,8)
	*Alloprevotella*	577	0,6	8 (100)	0,4 (0,2–0,7)
	*p-1088-a5_gut_group*	576	0,6	8 (100)	0,4 (0,2–0,7)
	*UCG-009*	499	0,5	8 (100)	0,4 (0,1–0,6)
	*UCG-002*	496	0,5	8 (100)	0,4 (0,2–0,5)
	*Odoribacter*	423	0,4	8 (100)	0,3 (0,2–0,4)
	*Rikenella*	258	0,3	8 (100)	0,2 (0,1–0,3)
	*Bacillus*	213	0,2	8 (100)	0,2 (0,1–0,2)
	*Terrisporobacter*	206	0,2	8 (100)	0,1 (0–0,2)
	*Agathobacter*	179	0,2	7 (87,5)	0,1 (0–0,2)
	*Oscillibacter*	177	0,2	7 (87,5)	0,1 (0–0,4)
	*GCA-900066575*	145	0,2	7 (87,5)	0,1 (0–0,2)
	*Pseudoflavonifractor*	143	0,1	6 (75)	0,1 (0–0,2)
	*Incertae_Sedis*	141	0,1	8 (100)	0,1 (0,03–0,2)
	*Flexilinea*	136	0,1	6 (75)	0,1 (0–0,2)
	*Dorea*	133	0,1	6 (75)	0,1 (0–0,2)
	*Mogibacterium*	117	0,1	7 (87,5)	0,1 (0–0,2)
	*Solobacterium*	113	0,1	6 (75)	0,1 (0–0,2)
	*Saccharofermentans*	106	0,1	6 (75)	0,1 (0–0,2)
	*Erysipelotrichaceae_UCG-009*	89	0,1	6 (75)	0,1 (0–0,1)
	*Papillibacter*	88	0,1	7 (87,5)	0,1 (0–0,1)
**Milk core microbiota**	*UCG-005*	4948	7,6	8 (100)	4,4 (1,3–8,5)
	*Bacteroides*	2652	4,1	8 (100)	2,4 (0,6–4,7)
	*Rikenellaceae_RC9_gut_group*	2638	4,0	7 (87,5)	2,3 (0–6,3)
	*Christensenellaceae_R-7_group*	2163	3,3	7 (87,5)	1,8 (0–2,7)
	*Prevotellaceae_UCG-004*	2049	3,1	7 (87,5)	1,7 (0–7,4)
	*Alistipes*	1593	2,4	8 (100)	1,5 (0,4–4,1)
	*Akkermansia*	1551	2,4	7 (87,5)	1,3 (0–3,6)
	*Fusobacterium*	1512	2,3	7 (87,5)	1,7 (0–3,2)
	*Pseudomonas*	1438	2,2	8 (100)	2,6 (0,3–13,4)
	*Monoglobus*	1362	2,1	8 (100)	1,4 (0,5–2,8)
	*Rhodococcus*	956	1,5	7 (87,5)	0,8 (0–1,9)
	*NK4A214_group*	934	1,4	8 (100)	1,0 (0,3–1,9)
	*Acinetobacter*	737	1,1	7 (87,5)	1,0 (0–4,0)
	*Phascolarctobacterium*	734	1,1	7 (87,5)	1,2 (0–6,0)
	*Porphyromonas*	719	1,1	8 (100)	0,8 (0,1–1,8)
	*Sphingomonas*	656	1,0	6 (75)	1,0 (0–3,3)
	*Escherichia/Shigella*	627	1,0	7 (87,5)	0,9 (0–3,9)
	*dgA-11_gut_group*	611	0,9	6 (75)	0,5 (0–2,2)
	*Nocardioides*	595	0,9	6 (75)	0,6 (0–2,2)
	*Treponema*	563	0,9	6 (75)	0,5 (0–1,2)
	*Iamia*	341	0,5	6 (75)	0,3 (0–0,8)
	*Luteimonas*	318	0,5	6 (75)	0,3 (0–0,7)
	*Family_XIII_AD3011_group*	317	0,5	6 (75)	0,3 (0–0,5)
**Blood core microbiota**	*Anaplasma*	69114	74,3	8 (100)	58,9 (5,3–92,3)
	*Mycoplasma*	1894	2,0	8 (100)	2,1 (0,3–7,8)
	*Rikenellaceae_RC9_gut_group*	1829	2,0	7 (87,5)	1,6 (0–5,3)
	*Bacteroides*	1682	1,8	6 (75)	1,5 (0–4,2)
	*Pseudomonas*	1505	1,6	8 (100)	1,4 (0,03–4,1)
	*Alistipes*	1255	1,3	6 (75)	1,2 (0–3,4)

A total of 43 taxa formed the core microbiota of faeces out of the 98 which were characterized. The core microbiota accounted for 96,8% of the relative abundance of the taxa obtained among faecal samples, displaying some level of homogeneity. Among these, 32 taxa were prevalent across 100% of the faecal samples ranging between 0,1–26,6% in relative group abundances. The taxa were constituted of typical gut microbes including *Romboutsia* which was the most abundant among faecal samples, recorded at 26,6% relative group abundance; followed by *UCG-005* (8,9%) and *Prevotellaceae_UCG-004* (8,3%). Similarly, microbes of interest such as *Monoglobus* and *Akkermansia* were both detected across 100% of the faecal samples at a relative abundance of 6,0% each. The microbial profile based on the most abundant taxa (15) present in 100% of the faecal samples was similar with minor variations in abundance as can be seen in Fig 5A.

A total of 23 out of the 374 genus ranking taxa obtained in milk formed the core microbiota, accounting for 45,9% relative group abundance. Only seven of these could be detected in 100% of the milk samples including among others *Bacteroides*, *Alistipes* and *Pseudomonas*, ranging in relative abundances between 1,1–7,6%. Taxonomic composition varied dramatically between milk samples based on the profile of the 15 most abundant taxa (Fig 5B). While majority of the abundant taxa were distributed throughout the samples, their proportions differed quite considerably.

Among blood samples, the core microbiota consisted of 6 taxa including *Anaplasma* (74,3%), *Mycoplasma* (2,0%), *Rikenellaceae*_RC9_gut_group (2,0%), *Bacteroides* (1,8%), *Pseudomonas* (1,6%) and *Alistipes* (7,3%) out of the 120 characterized genus-level taxa. These accounted for 83% of the relative abundance of the taxa obtained in blood. Only three genus-level taxa were detected across 100% of the blood samples *i*.*e*. *Anaplasma*, *Mycoplasma* and *Pseudomonas*. Taxonomic profiles of blood samples were marked by varying patterns of presence and absence of taxa throughout. Majority of the blood samples were dominated by *Anaplasma* as can be seen in Fig 5C.

**Fig 6:**
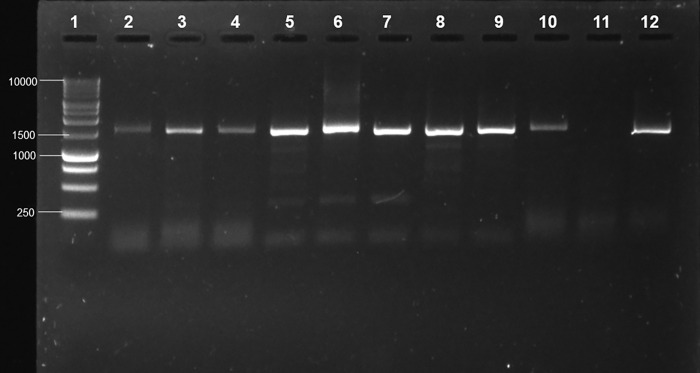
Gel electrophoresis of *Anaplasma* PCR targeting the 16S rRNA gene from blood samples. A: Lane 1 = 1 kb DNA ladder; 2–10 = *Anaplasma* positive samples; 11 = nuclease free H2O (-ve) control; 12 = *A*. *marginale* (+ve) control.

Although only detected among blood samples, *Anaplasma* was the single most abundant taxon overall. Thus, in order to obtain an overview of the prevalence of infection of cattle and the species involved in the sampled community, a total of n = 110 blood samples were screened by PCR, inclusive of samples from the lactating and non-lactating cows. The genus was detected from 65% (71/110) of the individual blood samples. The positive PCR amplicons can be seen in Fig 6 (S1 Raw images) at an expected fragment size of 1470 bp.

Characterization of the species by Sanger sequencing yielded 75% of the sequences that matched *A*. *marginale* (Accession numbers: AF414877 and KU686792) at 96,98–99,28% identity; while 25% matched *A*. *centrale* (Accession number: MF289480) at 97,87–98,29% identity on the NCBI database.

A number of commonly reported, potentially pathogenic taxa containing species of veterinary and / or medical significance were identified within and between the sample groups. Among faecal samples *Bacteroides*, *Bacillus*, *Prevotella*, *Streptococcus* and *Fusobacterium* were identified. From milk samples *Brucella*, *Helcococcus*, *Fusobacterium*, *Rhodococcus*, *Trueperella*, *Porphyromonas*, *Escherichia*/*Shigella*, *Streptococcus*, *Bacillus*, *Staphylococcus*, *Mycobacterium*, *Legionella*, *Klebsiella* and *Mycoplasma* could be identified. Noteworthy microbial taxa that could be identified in blood samples included *Bacillus*, *Escherichia/Shigella*, *Bartonella*, *Fusobacterium*, *Ehrlichia*, *Streptococcus*, *Peptostreptococcus*, *Prevotella*, *Rhodococcus*, *Klebsiella* and *Staphylococcus* in addition to the already mentioned *Anaplasma* and *Mycoplasma*. These taxa, together with their respective prevalences per sample group are listed under S5 Table.

### Shared and differentially abundant taxa across bovine faeces, milk and blood samples

We further identified taxa that were unique and shared between the three sample groups and plotted their intersection using UpSetR. A comparative analysis of the microbiota detected in each sample group was conducted to determine the extent of overlapping among them. A total of 15 of the 30 phyla (50%) were shared between faeces, milk and blood. Of the 243 microbial families detected, there were 49 which were shared between the three groups. A total of 26 families were exclusively shared between blood and milk; 3 between faeces and milk; and 1 family between faeces and blood (S2 Fig).

**Fig 7:**
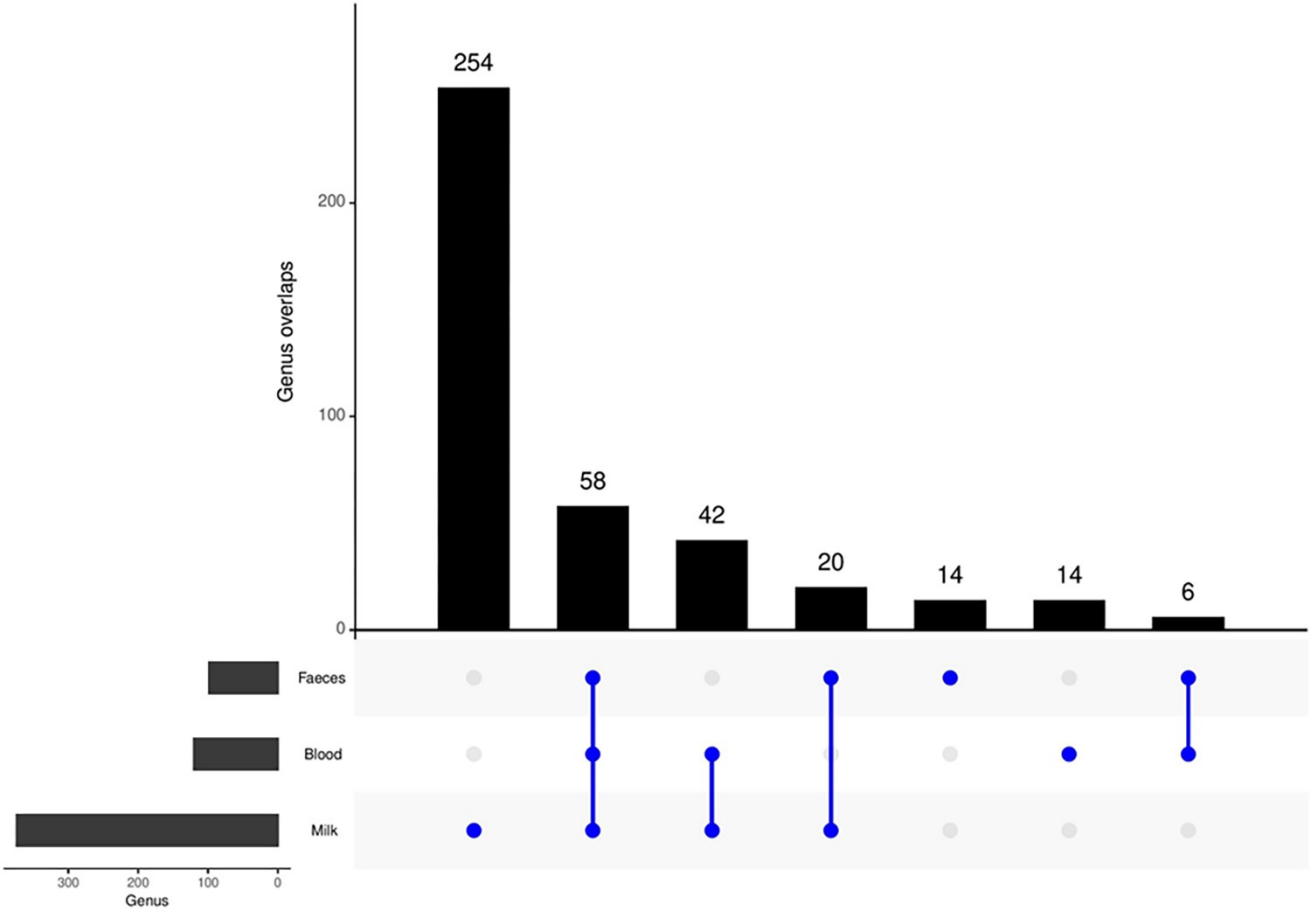
UpSetR intersection plot showing number of unique and shared ASVs at genus level between faeces, milk and blood groups.

Furthermore, from this analysis a total of 58 genus ranking taxa were found to be shared between the three sample groups accounting for 95,3%; 51,4%; and 18,6% of the taxa contained in faeces, milk and blood groups, respectively and 39,9% of the overall relative abundance (Fig 7). These taxa and associated classification, raw and relative abundances are listed in S6 Table. They were largely dominated by members of Firmicutes (n = 33; 57%) and Bacteroidota (n = 10; 17%). Of the 58 shared taxa across the three body sites, several important genera were identified and they include among others *Bacillus*, *Streptococcus*, *Akkermansia*, *Romboutsia*, *Fusobacterium* and *Bacteroides*. At the intersection between blood and milk, important genera such as *Mycoplasma*, *Escherichia/Shigella*, *Porphyromonas*, *Staphylococcus*, *Campylobacter*, *Klebsiella* and *Peptostreptococcus* were identified; while at the intersection between blood and faeces, *Prevotella* could be seen (S6 Table).

The blood and milk groups shared more taxa at genus level (100 genera in common, with 42 exclusively shared between the two groups) than faeces and milk (78 genera in common, with 20 exclusively shared) and faeces and blood (64 genera in common, with 6 exclusively shared), as shown in Fig 7 and S7 Table. Retrospectively, these findings corroborate the α-diversity analysis which showed that there were no significant differences between the microbial communities in blood and milk groups for all used index estimators. They however seem to contradict the findings observed through stacked bar plots and β-diversity analysis which showed that faeces and milk groups were more similar to each other than to blood however, it is important to note that in this analysis the whole number of taxa present per sample group and their identities were used to analyse the similarities between groups, rather than the relative abundances of dominant taxa which were used when creating stacked bar plots and in the estimation of β-diversity.

Although 58 genus-level taxa were shared between the three sample groups, these were represented by multiple ASVs which were mainly exclusively present in either faeces, milk or blood but rarely occurring concurrently across the sampled body sites. Those that did occur concurrently were representatives of 15 genus-level taxa *i*.*e*. *Romboutsia* (*e*.*g*. ASV49, ASV80); *UCG*-*005* (*e*.*g*. ASV2034, ASV630); *Prevotellaceae_UCG-004* (*e*.*g*. ASV2727, ASV2935); *Rikenellaceae_RC9_gut_group* (*e*.*g*. ASV1961); *Bacteroides* (*e*.*g*. ASV12295, ASV2471); *Christensenellaceae_R-7_group* (*e*.*g*. ASV1961); *Turicibacter* (*e*.*g*. ASV10387); *Fusobacterium* (*e*.*g*. ASV160); *Alistipes* (*e*.*g*. ASV1907, ASV7566); *Akkermansia* (*e*.*g*. ASV2594, ASV1343); *Prevotellaceae_UCG-003* (*e*.*g*. ASV976, ASV5359)*; dgA-11_gut_group* (*e*.*g*. ASV1250); *Phascolarctobacterium* (*e*.*g*. ASV1235, ASV2614); *Coprococcus* (*e*.*g*. ASV9068) and *Mailhella* (*e*.*g*. ASV5380). Bacterial signatures (at ASV level) representing the former nine genus-level taxa were detected concurrently in at least a single group of animals constituting a sample set across all three sample groups. The other concurrently occurring ASVs were randomly distributed across samples from the three sample groups.

**Fig 8:**
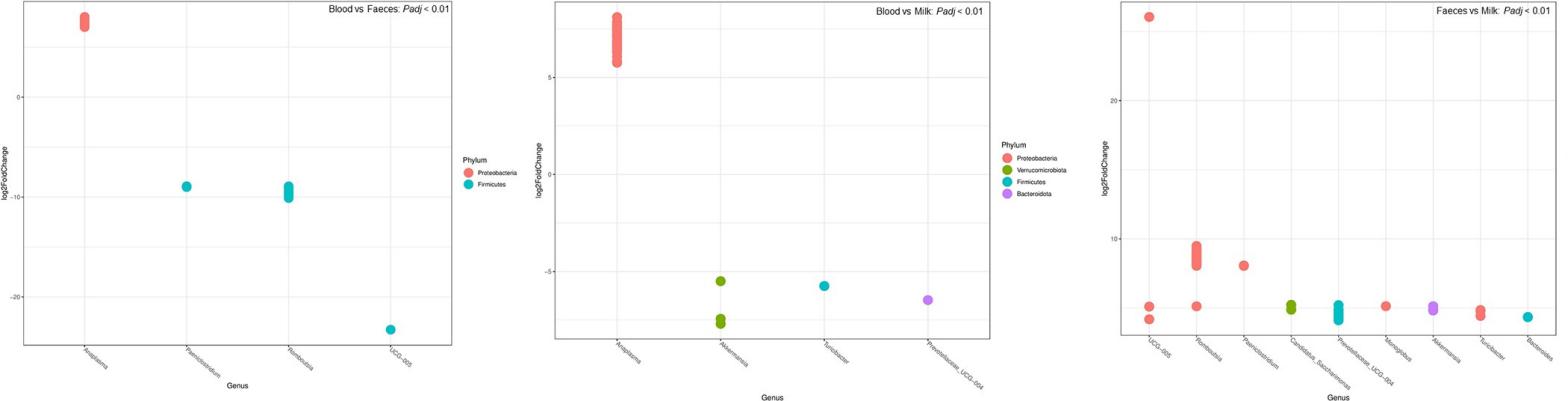
A: Differentially abundant genus-level taxa (*Padj* < 0,01) between blood and faeces. Positive log2-fold change indicates increased abundance in blood compared to faeces, negative log2-fold change indicates decreased abundance. The dots are ASVs representing genera. B: Differentially abundant genus-level taxa (*Padj* < 0,01) between bovine blood and milk. Positive log2-fold change indicates increased abundance in blood compared to milk, negative log2-fold change indicates decreased abundance. The dots are ASVs representing genera. C: Differentially abundant genus-level taxa (*Padj* < 0,01) between bovine faeces and milk. Positive log2-fold change indicates increased abundance of the genera in faeces compared to milk. The dots are ASVs representing genera.

In order to determine the compositional differences between the sample groups, analysis of differential abundance on DESeq2 normalized data was performed and several ASVs that varied significantly in their relative proportions with respect to sample groups were identified and reported significant at *Padj* < 0,01 (Fig 8A–8C; S8A–S8C Table). When analysed at the phylum and genus levels, the comparison between Blood vs Faeces; Blood vs Milk; and Milk vs Faeces identified five phyla and 18 genera; four phyla and four genera as well as four phyla and 15 genera displaying statistically significant differences, respectively.

Overall 602 ASVs (representing 18 genera) were found to be significantly DA between the blood and faecal sample groups. The 4 most discriminant taxa at genus level are plotted on Fig 8A (*Padj* < 0,01). *Anaplasma* (under phylum Proteobacteria) was significantly enriched in blood samples with an average log2-fold-change value of 6,93. This is line with the finding that *Anaplasma* could only be detected in blood. *Paeniclostridium* (- 8,40), *Romboutsia* (- 7,78) and *UCG-005* (- 6,56) under the phylum Firmicutes were greatly reduced in blood than in faeces, their respective average log2-fold-change values are indicated in parentheses.

Differences in abundance between blood and milk groups were defined by 53 ASVs representing four genus-level taxa which were significantly DA *i*.*e*. *Anaplasma* (Proteobacteria), *Akkermansia* (Verrucomicrobiota), *Turicibacter* (Firmicutes) and *Prevotellaceae*_*UCG-004* (Bacteroidota). *Anaplasma* was significantly enriched in blood (but absent in milk) with an average log2-fold-change value of 7,01 (*Padj* ≤ 0,01; Fig 8B), while *Akkermansia* (- 6,88), *Turicibacter* (- 5,75) and *Prevotellaceae*_UCG-004 (- 6,47) were significantly reduced in blood than in milk.

A total of 235 ASVs (representing 15 genera) were DA between faeces and milk. The differences in bacterial abundance between faeces and milk groups showed that the nine most discriminant taxa at genus level were all significantly enriched in faeces as opposed to milk (*Padj* < 0,01; Fig 8C). These taxa and their respective average log2-fold-change values included *UCG-005* (7,76), *Romboutsia* (7,43), *Paeniclostridium* (7,41), *Candidatus_Saccharimonas* (5,62), *Prevotellaceae_UCG-004* (5,19), *Monoglobus* (6,08), *Akkermansia* (5,57), *Turicibacter* (5,85) and *Bacteroides* (4,67). Genera such as *UCG-005*, *Romboutsia* and *Paeniclostridium* under the phylum Firmicutes were particularly overrepresented in faeces. Moreover, Firmicutes dominated the taxa (56%), followed by Bacteroidota (22%).

## Discussion

In this study we explored the microbial structure and composition of corresponding bovine faeces, milk and blood through sequencing of the V3-V4 hypervariable region of the 16S rRNA gene, employing the Illumina Miseq platform. This is an ideal platform for small-scale research due to its cost effectiveness, short turnaround time and comparatively high sequencing depth [50]. Examining the number of reads returned by sample type showed that they were disproportionate with the highest number recorded among faeces, followed by milk and blood. This was anticipated due to the difference in the type of samples being analysed, for instance, blood samples generally contain low microbial biomass while faecal samples contain high microbial biomass [51].

The DADA2 pipeline used to infer sequences was highly resolved with a total of 98,8% of all bacterial sequences found in faecal, milk and blood sample groups assigned at phylum level. Using ASVs to infer biological sequences has been reportedly found to allow for greater precision and reproducibility in taxonomic assignment compared to the use of operational taxonomic units (OTUs) [48]. Comparison of ASVs and OTUs in 16S rRNA sequence data analysis has previously shown that despite the larger number of OTUs generated as opposed to the number of ASVs from the same sequence data, similar trends could be seen in plots of observed OTUs and ASVs for alpha diversity analysis using Shannon and Simpson indexes [52]. However, the ability to distinguish sequence variants differing by as little as one nucleotide, imperceptible to OTU methods, makes inferring ASVs through DADA2 method preferable [41,52,53]. The precision of DADA2 improves downstream measures of diversity and dissimilarity and potentially allows amplicon methods to probe strain-level variation [41].

While a substantial amount of sequences could be classified at genus level, some could not and others were unaccounted for. Previous studies have reported many animal microbiomes containing certain proportions of unclassified bacteria at the same level. The reasons for this might be due to the limited database of 16S rRNA gene sequences and little research conducted on classification of animal microbiomes [54] or possibly due to the presence of reads with unclear sequence accuracy [55]. Furthermore, despite the high-resolution power of the pipeline, the 16S rRNA amplicon sequencing depth achieved in the current study was not sufficient for accurate taxonomic assignment at the species level. Although most of the genera had multiple unique ASVs associated with them, it was not possible to determine the species they each uniquely represented apart from only three taxa that were tentatively resolved to species level, representing a small fraction of the obtained sequences. However, not much could be drawn from these findings due to the scepticism surrounding the accuracy of species level-resolution from sequencing the V3-V4 hypervariable regions of the 16S rRNA gene, where resultant sequences of closely related species have been previously found to be 100% identical [56].

In order to establish the structure of the microbial communities with respect to the number of sequences representing taxonomic groups present and the distribution of abundances within the three sample groups, α-diversity was measured through Chao1, Shannon and Simpson indexes. The α-diversity estimates were all statistically significant between the three sample groups based on all the used index estimators. The α-diversity values of microbial communities contained in faecal samples tended to be higher compared to milk and blood samples, indicating greater microbial richness, abundance and evenness, at least at ASV level. Milk α-diversity values were also higher than those of blood. A study by Young *et al*. [18] revealed a similar observation where the faecal samples had greater microbial diversity than blood (macrophages) and milk (milk somatic cells) samples. Pairwise analysis between the groups showed that the microbial diversity varied significantly between blood and faeces as well as between faeces and milk groups, indicating that the observed number of ASVs and their abundance between these sample groups were not equally distributed. However, it did not vary significantly between milk and blood and this could be because many of the microbes contained within the milk and blood groups were similar in terms of their identities and there was some degree of homogeneity in their distribution between the two body sites. It has been previously suggested that the similarity between milk and blood microbiota of cows could be as a result of patrolling phagocytes in the mammary tissue that occasionally exit the bloodstream, traverse the epithelium, enter the mammary glands and eventually become shed in the milk however, this remains speculative [13].

Between group analysis on PCoA and NMDS plots were in agreement with the findings of the stacked bar plots, clustering samples by type. The distinct clustering indicates that the microbiota were markedly different especially with regards to some microbial populations that were only detected in either faeces, milk or blood. The PCoA plot indicated that microbial communities hosted within blood samples had a greater phylogenetic distance from milk and faecal communities with the exception of a few outliers. It further suggested that blood samples had a uniquely distinct microbial community compared to the other sample types analysed and that communities hosted in milk and faeces tended to have a shorter phylogenetic divergence with similar taxa and associated abundances. However, the most dominant taxa (in terms of abundance) influenced ordination on PCoA plots using weighted uniFrac metric despite the true picture of the overall number of taxa contained within and shared between samples. The analysis appeared to be sensitive to noise, *i*.*e*. samples containing unique ASVs in little abundances as well as those that contained fewer taxa in comparison to the rest. In contrast, NMDS on Bray distance metric was not affected by noise. Furthermore, the data obtained from both plots evidenced that sample type significantly influenced sample ordination, while farm origin and pooling strategy did not have any evident effect on sample clustering.

Although contaminant sequences were detected in the NTCs, retrospective analysis of contamination proved non-confounding to the findings of this study. The majority of the contaminant microbial genera have been previously detected in negative controls in a minimum of two or more studies and are said to originate from various sources which include kits and reagents contaminated during manufacturing and commensals on laboratory personnel and equipment [51]. Despite their high α-diversity values, faecal samples contained the least number of taxa across all taxonomic ranks compared to milk and blood. The reason for this was that although there was greater ASV richness among faecal samples, individual taxa were represented by large clusters of ASVs thus resulting in lesser microbial diversity among these samples as opposed to milk and blood samples. Physiologically, it could be attributed to the harsh gut environment that possibly does not allow for microbial variety, while blood and milk may be ideal media for cultivation of microbes. Within group analysis of stacked bar plots revealed that faecal samples had a more balanced microbial profile with little variation, this was supported by the composition of the core faecal microbiota. In contrast, the high inter group variation and much lesser number of species forming part of the core microbiota of milk and blood samples may have been an indication that there was no typical milk and blood microbiota. Furthermore, the blood samples were distinctly dominated by one genus. The source of variation among the samples could be linked to a variety of intrinsic and extrinsic factors which include among others, the diet and composition of the gut microbiota [57,58]; infection and immune statuses of the animals [57] particularly in the case of arthropod-borne pathogens observed in blood; the stage of lactation; and exposure to exogenous sources such as bedding material and herd faeces per farm [12,59,60].

The most prevalent bacterial groups detected in the faeces and milk included members of the Firmicutes and Bacteroidota phyla; while bacterial sequences from blood were predominantly members of Proteobacteria, a similar observation to Young *et al*.’s findings [18]. It has been proven that the gastrointestinal tract (GIT) of calves is seeded before birth with a diverse array of microbiota, changing drastically post-partum and successively predominated by Firmicutes, Bacteroidota, Proteobacteria and Actinobacteriota in decreasing order of abundance post-weaning [14,18,23,27,57,61,62]. In contrast to popular reports however, the Firmicutes and Bacteroidota were succeeded by Verrucomicrobiota in this study, following a similar microbial distribution pattern to donkey gut microbiota reported by Liu *et al*. [63].

Generally, the distribution and proportions of microbial phyla in milk seem to vary per sampled group depending on whether the subjects are healthy or mastitic. Milk microbiota from clinically healthy cows has been found to be mostly predominated by Firmicutes, followed by Bacteroidota, Proteobacteria and Actinobacteriota as the main bacterial phyla in many studies reviewed by Derakhshani *et al*. [12], which is quite similar to the distribution pattern obtained in this study. In contrast, Pang *et al*. [38] reported Proteobacteria as the major phylum followed by Firmicutes, Bacteroidota, and Actinobacteriota in milk from both healthy and mastitic quarters. Without conducting mastitis tests, it is difficult to draw conclusions about the abundance and distribution of the obtained taxa and how they are linked to the health statuses of the sampled animals in this study. However, according to Maity and Ambatipudi [11], regardless of whether the mammary gland is healthy or diseased, the main bacterial phyla like Firmicutes, Proteobacteria, Bacteroidetes and Actinobacteria are always there to shape the structure of bovine milk microbiota.

The bovine blood microbiota obtained in the current study mainly grouped under Proteobacteria, Firmicutes, Bacteroidota and Verrucomicrobiota. In contrast, a previous study reported the predominance of Tenericutes, Proteobacteria and Firmicutes in blood derived from Holstein dairy cows [20]. All in all, members of the phyla Firmicutes, Bacteroidota and Proteobacteria appeared to be the common denominator shaping the microbiota of the studied body sites. Both Firmicutes and Bacteroidota are said to play vital roles in the health of ruminants. Firmicutes function to degrade fiber and cellulose, while Bacteroidota function to degrade carbohydrates and proteins, and facilitate the development of gastrointestinal immunity [64]. Proteobacteria on the other hand are thought to play a key role in preparing the gut of neonates and young animals for colonization by the strict anaerobes required for healthy gut function by consuming oxygen and lowering redox potential in the gut environment; although their reputation is often tarnished due to the notoriety of some of the members which are opportunistic pathogens [65]. These above-mentioned roles of the three phyla are mainly associated with the GIT however, their roles in other niches need further investigation.

We further assessed and categorized taxa obtained in this study and evaluated their potential effects on both the livestock and members of the Waaihoek community. The observed faecal microbiota represented a mixture of taxa containing known anaerobic gut microbes (*e*.*g*. *Clostridium_sensu_stricto_1*, *Romboutsia* and *Bacteroides*) [26,57]; initial gut colonizers or bacteria found in the intestine but typically present on other mucosae (*e*.*g*. *Streptococcus* and *Staphylococcus*) [66]; and bacterial genera with potential health effects on cattle and human hosts (*e*.*g*. *Bacillus* and *Clostridium_sensu_stricto_1*) [26].

The milk samples generally consisted of a diverse range of opportunistic, commensal and pathogenic genera. These include frequently identified bacterial groups across the udder such as lactic acid bacteria (*e*.*g*. *Lactobacillus*) [12]; psychotrophic Gram-negative and -positive bacteria (*e*.*g*. *Pseudomonas* and *Bacillus*, respectively) [67,68]; skin-associated bacteria (*e*.*g*. *Staphylococcus* and *Corynebacterium*) [11,66]; and a number of taxa which are responsible for environmental and contagious mastitis (*e*.*g*. *Mycoplasma* and *Klebsiella*) [38,69]. Aside from the typical microbes that infect the mammary glands, the most abundant genus-level taxa in milk included gut associated taxa such as *UCG-005* (7,6%); *Bacteroides* (4,1%) and *Rikenellaceae*_RC9_gut_group (4,0%) which may have been endogenously translocated from the gut to the mammary glands or possibly breached the teat canal and gained access to the cistern from the environment.

The majority of the taxa observed in blood were atopobiotic, having possibly entered the bloodstream from their usual sites of colonization such as the gut (*e*.*g*. *Rikenellaceae_RC9_gut_group* and *Prevotella*); teats (*e*.*g*. *Streptococcus* and *Klebsiella*) [12,13]; uterus (*e*.*g*. *Fusobacterium* and *Bacteroides*) [20] or possibly inoculated into the bloodstream from an external source (*e*.*g*. *Ehrlichia* and *Bartonella*) [70].

Additionally, the important taxa obtained in this study could be further summarized under four categories of pathogens *i*.*e*. 1) arthropod-borne (*e*.*g*. *Anaplasma*, *Bartonella* and *Ehrlichia*) [70]; 2) food-borne and zoonotic (*e*.*g*. *Bacillus*, *Brucella*, *Campylobacter*, *Clostridium*, *Escherichia/Shigella*, *Klebsiella*, *Mycobacterium*, *Rhodococccus* and *Staphylococcus*) [11,71]; 3) mastitogenic (*e*.*g*. *Corynebacterium*, *Escherichia/Shigella*, *Klebsiella*, *Pseudomonas*, *Streptococcus*, *Staphylococcus* and *Mycoplasma*) [11,12,68,69]; and 4) metritic and abortigenic (*e*.*g*. *Bacteroides*, *Brucella*, *Fusobacterium*, *Helcococcus*, *Porphyromonas*, *Prevotella* and *Trueperella*) [20,71]. The majority of the taxa could fit under more than one category and they were found across all sampled body sites, except for arthropod-borne bacteria which were mainly restricted to the blood.

Important to note from this analysis is that the group of animals used in this study were possibly diseased. The abundance and distribution of various types of microbes in different proportions within samples may be associated with particular disease microbial dysbiosis. It has been reported that the suppression and / or over colonization of certain microbes in a particular niche results in disease pathogenicity, thus emphasizing the need to understand the interaction between the host environment and its inhabiting microbes [60]. Furthermore, since microbes with zoonotic potential were detected, precaution should be taken to prevent human infection in the sampled community. The routes of infection can be through consumption of contaminated meat and milk; via aerosol due to the proximity of the animal enclosures to their homes; occupational exposure through handling of infected animals as well as aborted foetal material [11]; and most importantly through the unsanitary practices associated with the use of cattle products and by-products by this rural community [7].

The genus-level taxa shared between the sample groups in our study accounted for a substantial amount of the overall relative abundance. A pattern of mutual exclusion of ASVs representing the shared bacterial taxa was observed between the sample groups. That is to say, they appeared to have distinct ecological relationships, with particular groups of ASVs occurring only in one sample type (*e*.*g*. faeces) and other groups in other sample types (*e*.*g*. milk or blood). We speculate that the distinctness of the ASVs representing bacterial taxa could possibly be attributed to their adaption to the respective host niches or it could be different species or variants of the same microbe. The simultaneous occurrence of 16S rRNA bacterial fragments originating from the GIT in the blood and milk samples of cows is suggestive of the presence of some endogenous route of transfer of microorganisms from the gut to the mammary glands via the bloodstream of cows as previously hypothesized [18]. However, testing of the viability of these microbes through culture and isolation across the body sites is necessary in order to determine if the gut is truly the source of viable microbial populations to the other sites [36]. Our findings therefore do not provide definite proof of the existence of this endogenous entero-mammary pathway, but warrant further investigation into the mechanisms and cells that allow the simultaneous occurrence of certain microbes in the sampled body sites.

The observed bacterial signatures that concurrently occurred in three body sites in corresponding samples did not match those that were previously reported at OTU level from at least one animal in a previous study [18], *i*.*e*. *Ruminococcus* and *Bifidobacterium* genera and an unclassified member of the Peptostreptococcaceae family. Owing to the pooling factor in our study, it could not be ascertained that the matching sequence variants across the body sites had originated from one animal, but it also does not dispel the possibility of this occurrence. Our results have the element of biological replication and by pooling we minimised the amount of information that could have been lost below the detection threshold when using individual samples as previously explained [72].

Discriminant analysis using DESeq2 served as an important tool to harvest the data generated by sequencing and to identify the bacterial genera that were significantly DA between bovine faeces, milk and blood (*Padj* < 0,01) for further analysis in our study. The majority of the DA taxa, except for *Anaplasma*, were detected across the three sample groups but greatly enriched in faeces than in milk and blood. This came as no surprise as *Romboutsia*, *Paeniclostridium*, *Akkermansia*, *Monoglobus*, *Turicibacter*, *Bacteroides*, *UCG-005*, *Candidatus_Saccharimonas*, and *Prevotellaceae_UCG-004* are typically gut-associated microbes [57,62,63].

*Anaplasma* was the most abundantly detected microbe in the entire analysis despite being present only among the blood group. The genus consists of tick-borne obligate intracellular organisms found exclusively within membrane-bound vacuoles in the cytoplasm of both vertebrate and invertebrate host cells [73]. In the current study,16S metagenomic sequencing seemed to be more sensitive than PCR in the detection of *Anaplasma* with 100% prevalence obtained in contrast to 65% achieved by PCR. However, PCR coupled with Sanger sequencing enabled characterization of the species to *A*. *marginale* and *A*. *centrale*, identical to field derived strains on the NCBI database. Both species cause bovine anaplasmosis with the less pathogenic species, *A*. *centrale*, being presently used as a live vaccine in many countries including RSA [73–75]. A recent high throughput sequencing based study conducted on bovine blood in this country similarly reported high proportions of *Anaplasma* (96,8% of total sequences excluding rare ones) characterized to a variety of species including both *A*. *marginale* (54%) and *A*. *centrale* (1%) as well as other important species such as *A*. *platys* and *A*. *phagocytophilum* among others [70]. This highlights the significance of characterizing the species of *Anaplasma* from bovine blood as it could be indicative of an active infection and some species within the genus are of zoonotic significance.

*Paeniclostridium* together with *Romboutsia* were reportedly the two largest genera in heifers of Holstein-Fresian breed in another study and were correlated to their digest functions and probably their physiological traits [57]. Likewise, in our study the pair was significantly enriched in faeces, with *Romboutsia* being the largest genus among faecal samples. The majority of *Romboutsia*-associated 16S rRNA gene sequences are said to have an intestinal origin, having been previously characterized from intestinal contents (*i*.*e*. duodenum, jejunum, ileum, colon and rectum) and faecal samples of various mammals including cattle [66,76]; as well as from milk and teat skin of dairy cattle [77]. *Romboutsia* species are known to cover a broad range of metabolic capabilities with respect to carbohydrate utilization, fermentation of single amino acids, anaerobic respiration and utilization of metabolic end products [76]. *Paeniclostridium* has also been recently detected in pasteurized milk where it was negatively correlated with the flavour substances which affected the quality and characteristics of the milk products [78].

Previous studies reported *Akkermansia* with relative abundances ranging between 0,56–8,64% in cow faecal samples [26], comparable with the findings of the current study (3,1–6,4%) among faecal samples. It’s been found in young calves and thought to play an opportunistic role as the microbe was detected in trace amounts or not detected at all in older animals previously [79], which is in contrast to our findings. The single species genus (*i*.*e*. *A*. *mucinophila*) is said to contribute to a healthy mucus-associated microbial composition and could also be used to prevent obesity and type 2 diabetes [63,80,81]. It was found to be an indicator of healthy breast milk in humans and presumed to confer beneficial functions as a probiotic [82]. Therefore, its abundance in the studied body sites may be important for the health of cows.

*Monoglobus*, a newly described genus in the family Monoglobaceae is also a single species genus (*M*. *pectinilyticus*), first isolated and described from human faeces [83]. It was unexpected among bovine faecal samples not to mention blood and milk samples, thus prompting further investigation into its occurrence in our study. Comparing findings from an earlier version of the taxonomic assignment tool (SILVA v.132) to those obtained in the newer version (SILVA v.138), it was found that top ranking unclassified taxa in the family Ruminococcaceae *i*.*e*. *Ruminococcaceae*_*UCG-010*, *Ruminococcaceae*_*UCG-013* and *Ruminococcaceae*_*UCG-014* were subsequently replaced by the genus *Monoglobus* in the newer version. As far as possible, our search did not yield a record of *Monoglobus* among the microbiota of bovine faeces until recently [27] where the authors tentatively identified a member of the family Oscillospiraceae (*i*.*e*. *UCG-005*) as being 93.7% similar to *M*. *pectinilyticus* in faecal samples of beef cattle. Thus, its role in the sampled body sites remains a mystery. In contrast to this however, members of Ruminococcaceae family are typically very common and reported to occur among the most abundant taxa found in the bovine gut microbiota and in faeces, highlighting their role in digestion of fiber and break down of complex carbohydrates [54,58].

Similarly to Young *et al*.’s findings, *Turicibacter* was also detected across all three body sites in our study [18]. The genus has been previously found to be dominant and significantly enriched in many parts of the GIT including the ileum, lumen and large intestine [62]. It was recently reported among the most representative genera in faecal samples of heifers and lactating cows [57] and has also been detected in high abundances in milk of dairy cattle [77]. The Gram-positive anaerobe is said to have significant functions such as providing anti-obesity effects, reducing metabolic stress, and inhibiting inflammatory reactions however, its metabolism and interaction with the host in the gut are still unclear [81].

*Bacteroides* spp. are well-known intestinal bacteria that can be both beneficial and harmful to their host. The genus was reported for the first time in cow milk microbiota in 2013 [31], subsequent studies have also characterized it from milk [18,28] including the current. We have also detected the bacterium among the blood samples, ranking fifth in terms of abundance. The bacterium was found to be notably high in abundance in new-born calves as opposed to older animals by Jami *et al*. [79]. Which could be correlated to its described vital role in the development of immunological tolerance to commensal microbiota and participation in natural genetic transfer of antimicrobial resistance genes to neonates [23,26]. Therefore, its high abundance particularly in the milk samples of lactating cows might be for the purpose of imparting immunity to the suckling calves.

Various unclassified bacteria (at genus level) derived from Oscillospiraceae (*i*.*e*. *UCG-005*); Saccharimonadaceae (*i*.*e*. *Candidatus_Saccharimonas*); and Prevotellaceae (*i*.*e*. *Prevotellaceae*_*UCG-004*) families were among the most discriminant taxa and were significantly DA between the three body sites. These unclassified fragments have been reported in a variety of ruminant NGS-based studies [18,27,62,84]. According to Huws *et al*. [84], they may play a predominant role in ruminal biohydrogenation. Their frequent reporting and appearance in high abundances in the current and other studies signifies the importance of research based on classification of bacterial taxa and updating the database of 16S rRNA gene sequences found in the gut (and ultimately faeces), milk and blood including their roles therein.

## Conclusion

In conclusion, characterization of the microbiota of faeces, milk and blood from cows using high throughput sequencing of the V3-V4 hypervariable region of the 16S rRNA gene provided new insights into the microbial structure and composition of the investigated body sites individually and in common, particularly in the South African context. The concurrent detection of microbes across the three sample groups can potentially contribute towards knowledge acquisition regarding the hypothesized endogenous entero-mammary pathway in ruminants.

DADA2 inference of ASVs was highly resolved and it was efficient for the purpose of the current study; however, species-level resolution was limited by the sequencing depth achieved. Improvement and manipulation of the available technologies to their fullest capacity, can yield more sequencing depth, define more bacterial taxa at the genus level and achieve greater species level resolution [55].

While the targeted hypervariable regions of the 16S rRNA gene may not be the optimal genomic regions for detecting the presence of pathogenic species, our findings indicate that this type of broad scale microbial survey may be useful in determining the presence of potential pathogens from an array of bacteria. This can in turn guide more targeted sampling and detection of both pathogenic and commensal bacteria across body sites.

Future studies are envisaged to investigate the functionality of the microbiota found in the studied body sites, their potential role in maintaining optimal health and the onset of disease and the mechanisms involved in the translocation of gut microbes into milk and blood. Furthermore, these studies should be designed with the ‘One-World, One-Health’ approach in mind in order to primarily aid in improvement of productivity through a better understanding of microbial function and ecology. Secondarily, to help decrease environmental pollution, contamination of food and dissemination of disease among animals and between animals and humans.

## Supporting information

S1 Fig A: Alpha diversity box-plots showing Chao1 richness estimates per sample group. *Significant at *P* < 0,05. B: Alpha diversity box-plots showing Shannon diversity estimates per sample group. *Significant at *P* < 0,05. C: Alpha diversity box-plots showing Simpson’s diversity estimates per sample group. *Significant at *P* < 0,05.
(TIF)

S2 Fig UpSetR intersection plot showing number of unique and shared taxa at family level between faeces, milk and blood groups. (TIF)

S1 Table Read counts tracked through the DADA2 pipeline including ASV counts, richness and genus level-resolved ASVs per sample. (XLSX)

S2 Table Alpha diversity values calculated using Shannon, Simpson and Chao1 indices. (XLSX)

S3 Table Total number of taxa detected per taxonomic rank across bovine faeces, milk and blood. (XLSX)

S4 Table Top 15 abundant taxa with their respective overall rankings and distribution across the three sample groups. (XLSX)

S5 Table Prevalence of potentially pathogenic genera of veterinary significance per sample group. (XLSX)

S6 Table Bacterial taxa shared between bovine faeces, milk and blood and their overall raw and relative abundances. (XLSX)

S7 Table Genus-level taxa exclusively detected and shared between faeces, milk and blood samples. (XLSX)

S8 Table A-C Differentially abundant taxa between blood and faeces; blood and milk and; faeces and milk (*Padj* < 0,01).
(XLSX)

S1 Raw images Gel electrophoresis image of *Anaplasma* PCR targeting the 16S rRNA gene from blood samples. Image taken under UV transillumination using Enduro™ GOS gel documentation system. Lane 1 = 1 kb DNA ladder; 2–10 = *Anaplasma* positive samples; 11 = nuclease free H_2_0 (-ve); 12 = *A*. *marginale* (+ve).
(PDF)
