# Supplementary figures and images for: Exploration and comparison of bacterial communities present in bovine faeces, milk and blood using 16S rRNA metagenomic sequencing
Source: PLoS One. 2022 Aug 31;17(8):e0273799. doi: 10.1371/journal.pone.0273799 (PMC9432762; doi:10.1371/journal.pone.0273799)

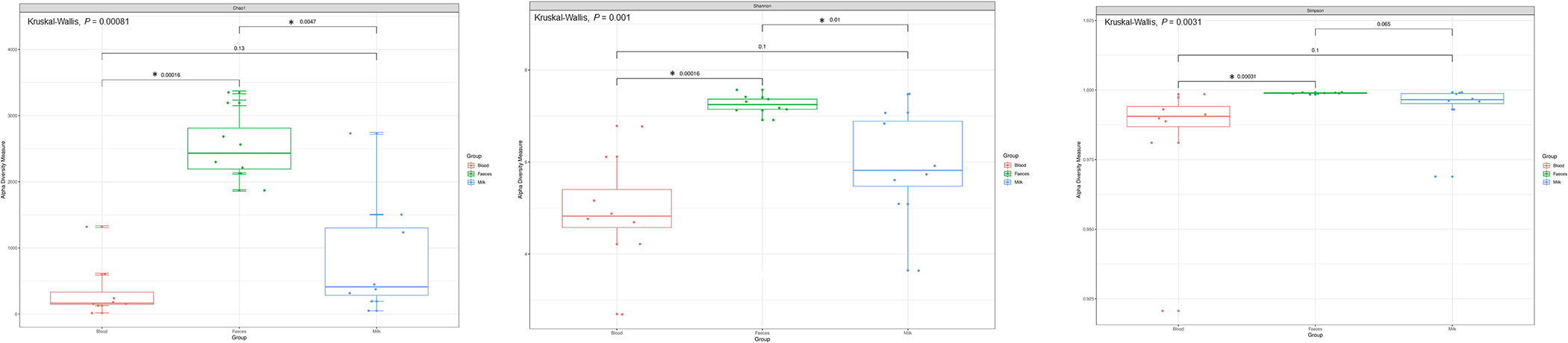

Supplement: S1 Fig — A: Alpha diversity box-plots showing Chao1 richness estimates per sample group. *Significant at P < 0,05. B: Alpha diversity box-plots showing Shannon diversity estimates per sample group. *Significant at P < 0,05. C: Alpha diversity box-plots showing Simpson’s diversity estimates per sample group. *Significant at P < 0,05. (TIF) [file pone.0273799.s001.tif]

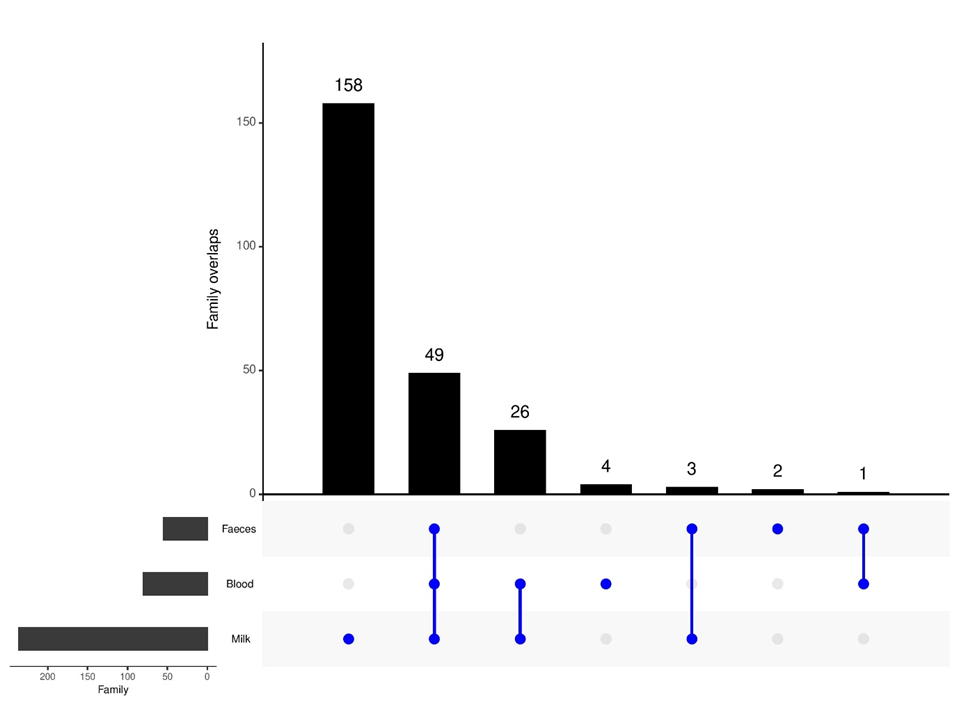

Supplement: S2 Fig — (TIF) [file pone.0273799.s002.tif]
