# Supplementary material for: Exploration and comparison of bacterial communities present in bovine faeces, milk and blood using 16S rRNA metagenomic sequencing
Source: PLoS One. 2022 Aug 31;17(8):e0273799. doi: 10.1371/journal.pone.0273799 (PMC9432762; doi:10.1371/journal.pone.0273799)
Supplement: S1 Raw images — Image taken under UV transillumination using Enduro™ GOS gel documentation system. Lane 1 = 1 kb DNA ladder; 2–10 = Anaplasma positive samples; 11 = nuclease free H20 (-ve); 12 = A. marginale (+ve). (PDF) [file pone.0273799.s011.pdf]

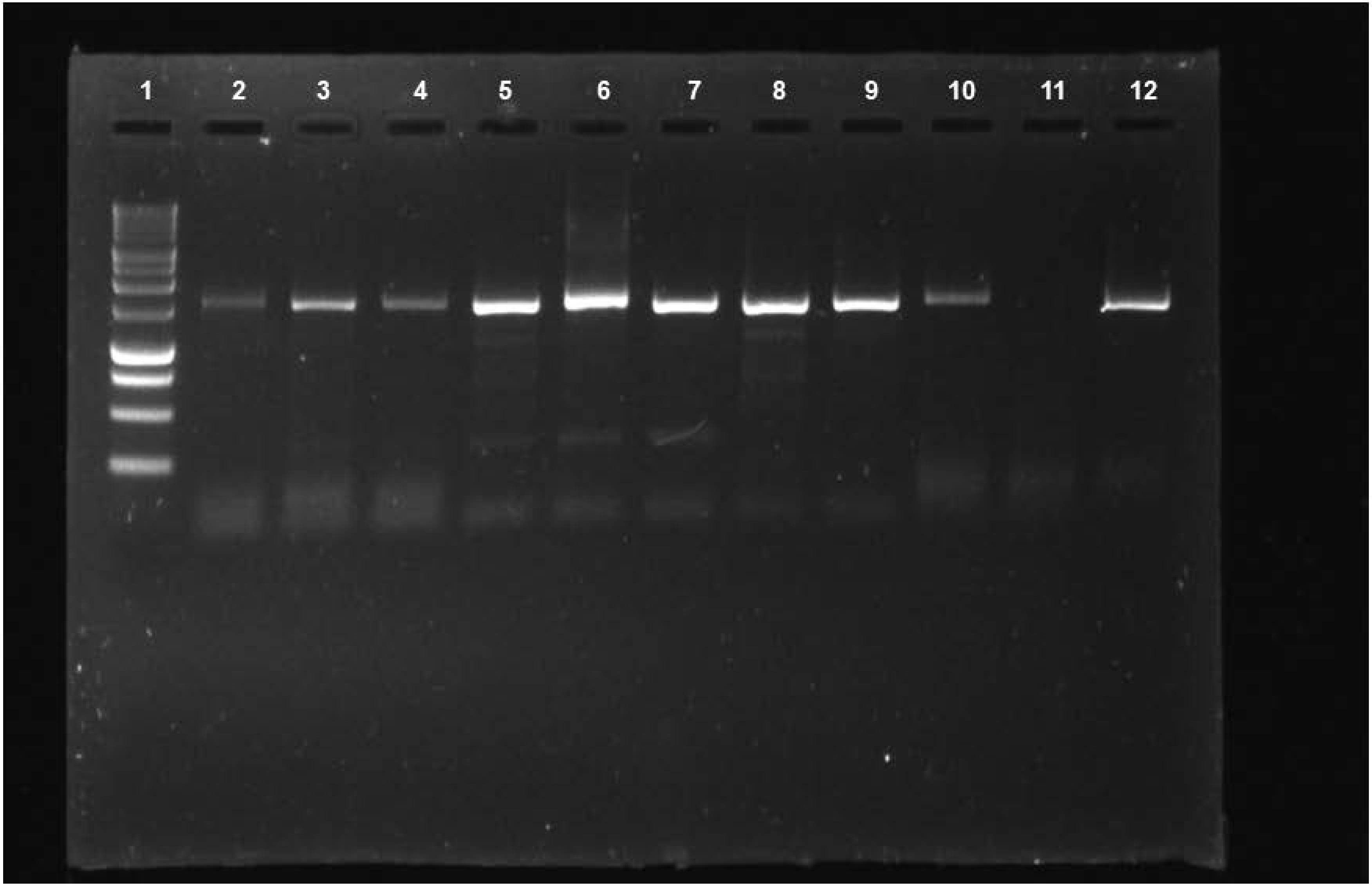

Fig 6: Gel electrophoresis image of *Anaplasma* PCR targeting the 16S rRNA gene from blood samples. Image taken under UV transillumination using Enduro™ GDS gel documentation system. Lane 1 = 1 kb DNA ladder; 2 - 10 = *Anaplasma* positive samples; 11 = nuclease free H_2O (-ve); 12 = *A. marginale* (+ve).
